# The gut microbiota of larvae of *Rhynchophorus ferrugineus* Oliver (Coleoptera: Curculionidae)

**DOI:** 10.1186/1471-2180-14-136

**Published:** 2014-05-30

**Authors:** Marcello Tagliavia, Enzo Messina, Barbara Manachini, Simone Cappello, Paola Quatrini

**Affiliations:** 1 Department STEBICEF, University of Palermo Viale delle Scienze Ed.16, Palermo 90128, Italy; 2 Istituto per l’Ambiente Marino Costiero (C.N.R. – IAMC) U.O.S. di Messina, Spianata S. Raineri, 86, Messina 98122, Italy; 3 Istituto per l’Ambiente Marino Costiero (C.N.R. – IAMC) U.O.S. di Capo Granitola, Via del Mare, 3 Torretta-Granitola, Mazara, TP 91021, Italy

**Keywords:** Enterobacteriaceae, *Dysgonomonas*, TTGE, Pyrosequencing

## Abstract

**Background:**

The red palm weevil (RPW) *Rhynchophorus ferrugineus* Olivier (Coleoptera: Curculionidae) is one of the major pests of palms. The larvae bore into the palm trunk and feed on the palm tender tissues and sap, leading the host tree to death. The gut microbiota of insects plays a remarkable role in the host life and understanding the relationship dynamics between insects and their microbiota may improve the biological control of insect pests. The purpose of this study was to analyse the diversity of the gut microbiota of field*-*caught RPW larvae sampled in Sicily (Italy).

**Results:**

The 16S rRNA gene-based Temporal Thermal Gradient Gel Electrophoresis (TTGE) of the gut microbiota of RPW field-trapped larvae revealed low bacterial diversity and stability of the community over seasons and among pools of larvae from different host trees. Pyrosequencing of the 16S rRNA gene V3 region confirmed low complexity and assigned 98% of the 75,564 reads to only three phyla: Proteobacteria (64.7%) Bacteroidetes (23.6%) and Firmicutes (9.6%) and three main families [Enterobacteriaceae (61.5%), Porphyromonadaceae (22.1%) and Streptococcaceae (8.9%)]. More than half of the reads could be classified at the genus level and eight bacterial genera were detected in the larval RPW gut at an abundance ≥1%: *Dysgonomonas* (21.8%), *Lactococcus* (8.9%), *Salmonella* (6.8%), *Enterobacter* (3.8%), *Budvicia* (2.8%), *Entomoplasma* (1.4%), *Bacteroides* (1.3%) and *Comamonas* (1%). High abundance of Enterobacteriaceae was also detected by culturing under aerobic conditions. Unexpectedly, acetic acid bacteria (AAB), that are known to establish symbiotic associations with insects relying on sugar-based diets, were not detected.

**Conclusions:**

The RPW gut microbiota is composed mainly of facultative and obligate anaerobic bacteria with a fermentative metabolism. These bacteria are supposedly responsible for palm tissue fermentation in the tunnels where RPW larvae thrive and might have a key role in the insect nutrition, and other functions that need to be investigated.

## Background

The red palm weevil (RPW) *Rhynchophorus ferrugineus* Olivier (Coleoptera: Curculionidae) is widely considered the most damaging insect pest of palms in the world, even in all the countries where it has been accidentally introduced [1]. RPW larvae feed within the apical growing point of the palms, producing a wet fermenting frass inside the tunnels [2], creating extensive damage to palm tissues and weakening the structure of the palm trunk; the resulting damage is often only visible long after infestation, when palms are close to death [3-5] (Additional file 1).

Insect intestinal tracts harbour rich communities of non-pathogenic microorganisms [6,7] and a single gut can harbour 10^5^–10^9^ prokaryotic cells [6] that have been affiliated to twenty-six phyla, at least for the insects studied to date [8]. It is increasingly evident that the microbiota of animals (humans included) plays a remarkable role in the host life. The genetic wealth of the microbiota affects all aspects of the holobiont’s (host plus all of its associated microorganisms) fitness such as adaptation, survival, development, growth, reproduction and evolution [9]. When not strictly essential for survival, the insect gut microbiota affects many aspects of host phenotype; it can increase the digestive efficiency of soluble plant polysaccharides [10,11] and can mediate interactions between the host and potential pathogens [12]. Recent work suggests that the gut microbiota not only provide nutrients, but is also involved in the development and maintenance of the host immune system. However, the complexity, dynamics and types of interactions between the insect hosts and their gut microbiota are far from being well understood [13]. Understanding the relationship dynamics between insects and their microbiota can improve the biocontrol of insect pests, which is a focus of much insect gut microbiology studies.

Despite the economic and environmental damages caused by the RPW in all the areas where it is endemic and where it has been accidentally introduced, little is known about its gut microbiota. The bacterial community that is embedded in the frass produced inside the tunnels of the palm *Phoenix canariensis* Chabaud by the RPW larvae is dominated by Enterobacteriaceae with a facultative fermentative metabolism [2].

The purpose of this study was to analyse the diversity of the gut microbiota of the *R. ferrugineus* larvae, that represent the development stage responsible for damages to palms. Field*-*caught larvae were sampled from its favourite host *P. canariensis* in different seasons and sites in Sicily (Italy), and analysed for the diversity of their gut microbiota. The analysis of the bacterial community was carried out by culture-independent methods using temporal thermal gradient gel electrophoresis (TTGE) and FLX454 pyrosequencing of PCR-generated amplicons from the 16S rRNA gene.

## Results

### Total diversity of the gut microbiota of field caught *RPW larvae*

**Figure 1:**
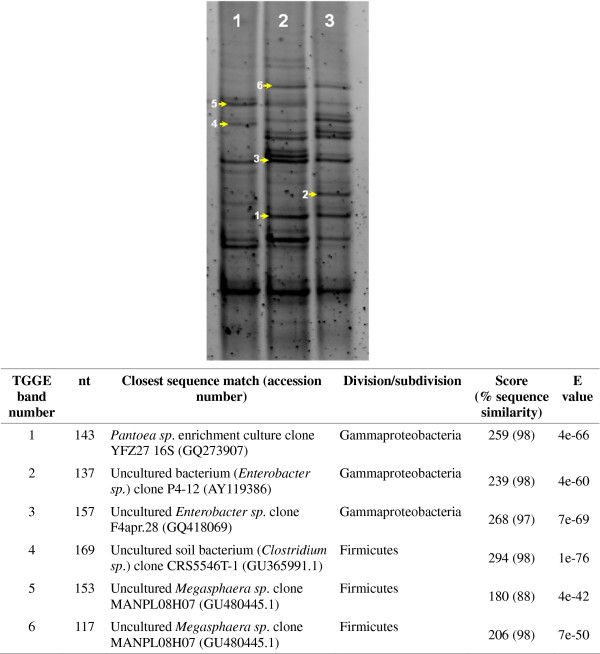
**Temporal Thermal Gradient gel Electrophoresis (TTGE) profiles of PCR-amplified 16S gene fragments derived from field collected larvae of *Rhynchophorus ferrugineus*.** Lane 1: TTGE profile of a pool of three larvae (average weight: 3.25 g; SD: 0.55) collected in December 2010 in a palm tree in the urban area of Palermo (Italy). Lane 2: TTGE profile of a pool of three larvae collected in April 2011 (average weight: 3.86 g; SD: 0.64) in the urban area of Palermo (Italy). Lane 3: TTGE profile of a pool of three larvae collected in April 2011 (average weight 3.60 g; SD: 0.53) in San Vito lo Capo (Trapani, Italy).

Bacterial TTGE profiles were generated using PCR-amplified bacterial 16S rRNA gene fragments from the content of pooled RPW larval guts collected from the trunks of infested *P. canariensis* palms in three different seasons and two areas in Sicily (Italy). TTGE band profiles indicate the presence of an average of 25 bands per sample, that correspond to putative bacterial phylotypes in RPW larval guts. An example of TTGE gel is shown in Figure 1, where three different pooled guts collected in December 2010 and April 2011 in Palermo (lanes 1 and 2, respectively), and in April 2011 in San Vito lo Capo (Trapani, lane 3) were analysed. All samples shared 16 bands, while 4, 2 and 4 bands were unique for samples 1, 2, 3, respectively. Similar profiles were obtained from larvae collected in October both in Palermo and Trapani (data not shown). Random sequencing of TTGE bands identified the presence of uncultured Gammaproteobacteria (of the genera *Pantoea* and *Enterobacter*) and Firmicutes (of genera *Megasphaera* and *Clostridium*) (Figure 1).

### Pyrosequencing

**Figure 2:**
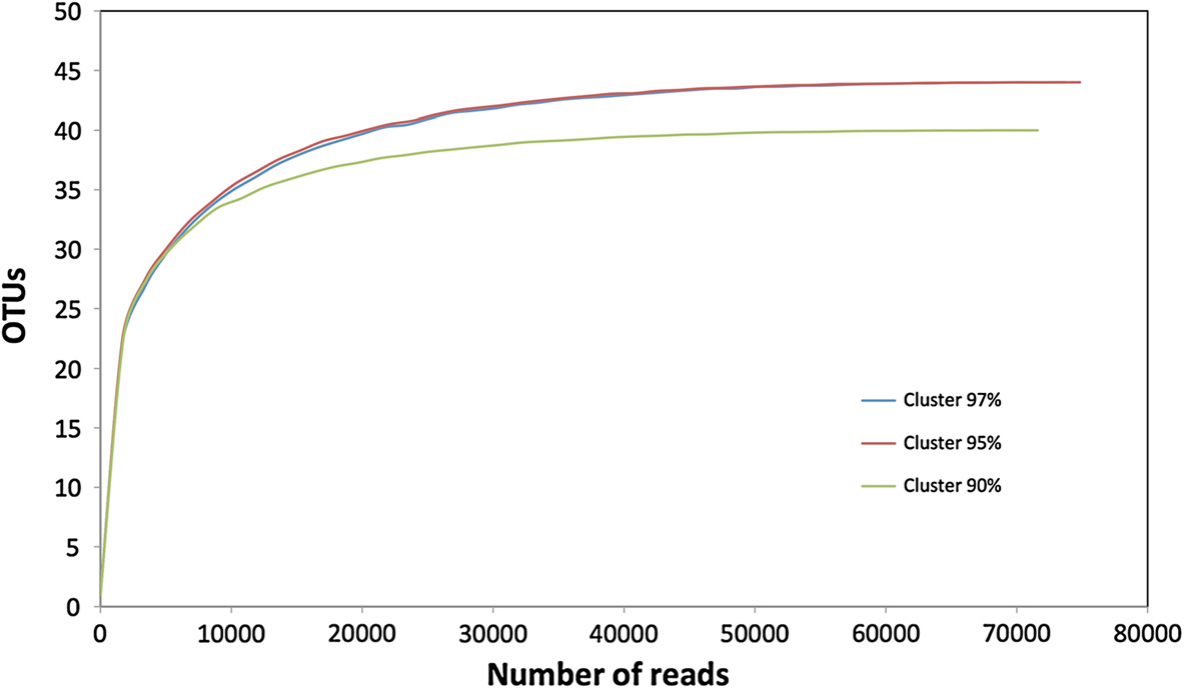
Rarefaction curves of OTUs clustered at different % ID in the gut of RPW larvae.

Pyrosequencing of the 16S rRNA gene amplicons was carried out on three pooled RPW larval guts sampled in Palermo in April 2011 (indicated as lot A). The analysis produced a total of 79,204 reads with an average length of 320.6 nucleotides that became, after quality filtering and clustering (needed for Ribosomal Database Project analysis), 75,564 for 97%, 76,724 for 95%, and 73,579 for 90% of similarity (Additional file 2). Reads were assigned to 41 operational taxonomic units (OTUs) at 90% of sequence identity threshold, and to 45 OTUs at 95% and 97% identity threshold, respectively, in order to perform rarefaction analysis. The total number of clusters obtained after filtering was of 2,107 (1,756 singletons) for 97%, 910 (530 singletons) for 95%, and 244 (124 singletons) for 90% of similarity, respectively. The rarefaction curves tended towards saturation at similar numbers of clusters at 97%, 95% and 90% pairwise ID thresholds (Figure 2). Subsequent analysis was, therefore, conducted at 97% ID.

**Figure 3:**
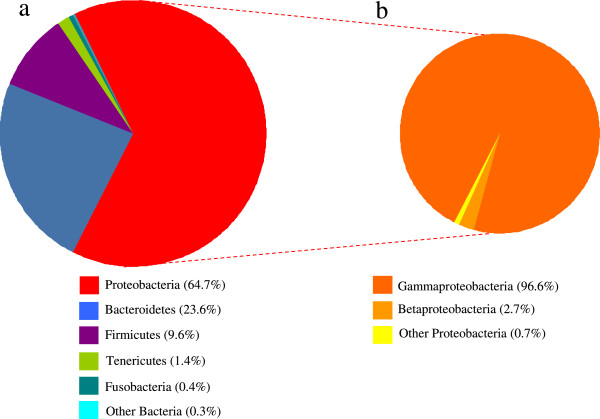
**Relative abundance of a) bacterial Phyla and b) classes of Proteobacteria in the gut of field caught RPW larvae as detected by pyrosequencing.** Values ≤ 0.1% are included in “other bacteria” (see Additional file 2).

**Figure 4:**
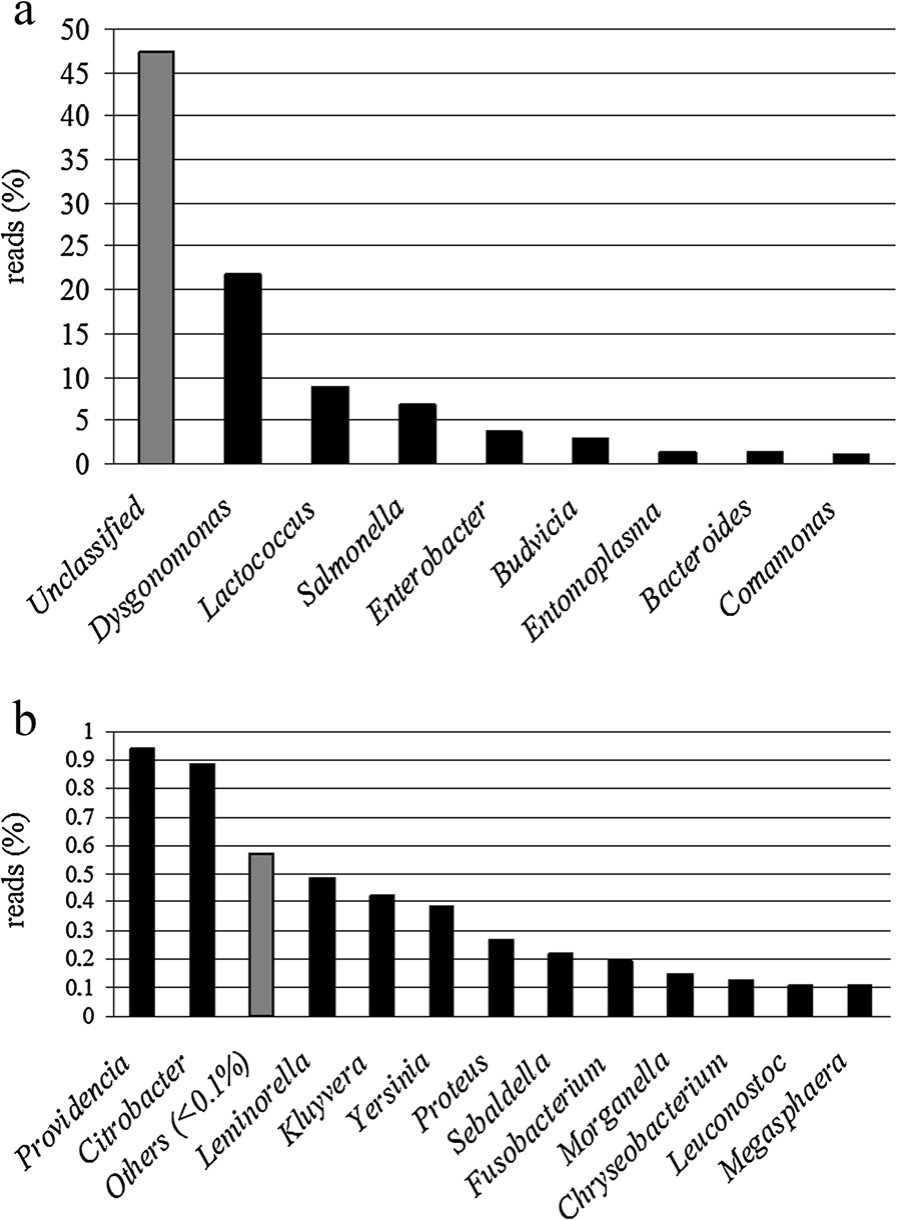
**Relative abundance of bacterial genera a) above 1% and b) below 1% in the gut of field caught RPW larvae as detected by pyrosequencing.** “Others” indicates 35 genera below 0.1% (see Additional file 2).

Only three phyla account for 98% of the reads: these are Proteobacteria (64.7%), Bacteroidetes (23.6%) and Firmicutes (9.6%); the remaining 2% is represented by Tenericutes (1.4%) Fusobacteria (0.4%) and other Bacteria (0.2%) (Figure 3a). Proteobacteria are mainly represented by Gammaproteobacteria (96.7%) followed by Betaproteobacteria (2.71%) (Figure 3b). More than 98% of the reads were classified at the family level, with Enterobacteriaceae representing the 61.5% of the assemblage, followed by Porphyromonadaceae (22.1%) and Streptococcaceae (8.9%) (Additional file 3). More than half of the reads (52.7%) could be classified at the genus level and eight bacterial genera were detected in the larval RPW gut at an abundance ≥1% (Figure 4a). *Dysgonomonas* sequences account for the 21.8% of the whole sequences and this is the most represented genus in the gut of RPW larvae, followed by *Lactococcus* (8.9%) *Salmonella* (6.8%), *Enterobacter* (3.8%), *Budvicia* (2.8%), *Entomoplasma* (1.4%) *Bacteroides* (1.3%) and *Comamonas* (1%). Other twelve genera are represented at a value between 1% and 0.1% (Figure 4b). The phylogenetic tree of 16S rRNA gene amplicons clustered at 97% consensus is shown in the Additional file 4.

### Diversity of cultivable bacteria

Bacterial isolation under aerobic conditions was carried out on three lots of three pooled RPW larval guts (lots A, B, C), all sampled in April 2011. The dilution plate counts on NA gave an average of 1.5 × 10^7^ CFU gut^-1^, without differences among the three pools. Forty-four phenotypically different isolates, obtained from the dilution plates of the three pooled guts independently, were grouped into ten OTUs (Table 1) by ARDRA analysis with the restriction enzymes *AfaI* and *AluI,* that gave five and seven different restriction profiles, respectively (data not shown). 16S rRNA gene sequencing of representative isolates assigned the cultivable bacteria to the families Enterobacteriaceae (68.2%), Bacillaceae (20.5%), Comamonadaceae (9%) and Xanthomonadaceae (2.7%) (Table 1). The genus *Citrobacter* is the most abundant among the isolates (29.55%), followed by the genera *Klebsiella* (20.45%), *Bacillus* (20.45%) and *Budvicia* (11.36%).

**Table 1:** **Phylogenetic affiliation of representative bacterial isolates from the gut of *R. ferrugineus *larvae as assigned by the Naïve Bayesian rRNA Classifier Version 2.4, of the Ribosomal Database Project II (RDP) and EMBL/SwissProt/GenBank non-redundant nucleotide database BLAST analysis**

**OTU**	**Phylum**	**Class**	**Family**	**N. of isolates in the OTU**	**Isolate**	**Most closely related sequence (MegaBLAST)**	**Genbank acc. N.**	**ID%**
A	Proteobacteria	Betaproteobacteria	Comamonadaceae	4	RPWA5.3	*Comamonas nitrativorans* strain 23310	NR025376.1	98
B		Gammaproteobacteria	Enterobacteriaceae	5	RPWA3.3	*Budvicia aquatica* strain Eb 13/82	NR025332.1	98
					RPWC1.3	*Uncultured bacterium* clone J44	GQ451198.1	99
C				10	RPWA2.8	*Citrobacter koseri* strain LMG 5519	HQ992945.1	99
D				3	RPWC2.4	*Citrobacter koseri* complete genome ATCC BAA-895	CP000822.1	99
E				1	RPWC1.2	Uncultured bacterium clone MFC4P_173	JF309179.1	99
F				9	RPWB1.1	*Klebsiella oxytoca* strain LF-1	EF127829.1	99
					RPWA1.1	*Klebsiella oxytoca* strain NFL28	GQ496663.1	99
					RPWA1.5	*Klebsiella sp*. 2392	JX174269.1	93
					RPWC4.3	*Klebsiella sp*. Co9935	DQ068764.1	99
G				1	RPWC2.2	*Proteus sp*. LS9(2011)	JN566137.1	99
H				1	RPWA1.6	*Salmonella enterica* subsp. arizonae serovar 62:z4,z23,	CP000880.1	99
I			Xanthomonadaceae	1	RPWC3.1	*Stenotrophomonas sp*. DD7	JQ435720	99
J	Firmicutes	Bacilli	Bacillaceae	9	RPWA4.1	*Bacillus muralis* strain cp5	JN082264.1	99
					RPWB1.3	*Bacillus sp*. 4014	JX566611	99
					RPWB1.4	*Bacillus sp*. DP5(2011)	JF825992.1	99
					RPWB3.2	*Bacillus megaterium* strain NBRC 12068	AB680229.1	99

Most of the sequences having homology with those of RPW isolates are from bacteria isolated from animals’ gut or from plants (endophytes), as well as from wastewater or bioremediation treatment plants and anaerobic marine sediments. Some of the *Citrobacter* and *Klebsiella* 16S rRNA sequences are almost identical to those from bacteria previously isolated from the frass produced by RPW larvae in the tunnels of palm trees (Additional file 5) [2].

Several attempts were made to surface-sterilize the larvae using different protocols; nevertheless the control plates, obtained by streaking on Nutrient Agar the cuticle of sterilized larvae, showed the growth of some colonies. Seven of these colonies were purified and analysed by ARDRA as described above. One representative isolate for each of the three OTUs obtained was chosen for 16S rRNA gene sequencing and all the three isolates were affiliated to the genus *Bacillus*, with their closest relatives in *B. thuringiensis *[14] and *B. aerophilus *[15] (Additional file 5).

Enrichment cultures were set for the isolation of acetic acid bacteria (AAB). AAB are known to establish symbiotic associations with the midgut of insects relying on sugar-based diets, such as nectars, fruit sugars, or phloem sap [16]. At the end of the incubation period, four CaCO_3_ dissolving colonies were isolated from the enrichment cultures and identified by 16S rDNA sequencing. Unexpectedly, all the isolates that were able to use sorbitol and to dissolve CaCO_3_ in the agar plates were assigned to the genus *Klebsiella* (Additional file 5).

## Discussion

In this study, the diversity of the gut microbiota of *Rhynchophorus ferrugineus* (RPW), collected on infested palm trees *Phoenix canariensis*, was first analysed by TTGE of the PCR-amplified bacterial 16S rRNA gene fragments. The TTGE profiles obtained from different lots of larvae, sampled in different seasons and geographical sites, show relatively low complexity (average of 25 OTUs) and high similarities regardless the site of sampling and season, suggesting that the composition of the RPW microbiota is stable over time and among pools of larvae from different host trees.

In order to identify the gut bacterial community of RPW larvae, the variable region 2 (V2) of the bacterial 16S rRNA gene, already successfully employed in the analysis of several microbial communities [17-19], was analysed by pyrosequencing. The analysis confirmed that the bacterial community of the RPW larvae has low diversity although, as expected, more OTUs were identified in respect to TTGE analysis. Contrasting results are reported for bacterial diversity of gut microbiota of other coleopterans; high diversity and complexity was observed among tree xylophagous beetles that rely on the microbiota for efficient lignocellulose metabolism and thus survival [8], while low diversity was recorded in the gut of the red turpentine beetle [20]. The RPW larvae are the major responsible for the palm damages because they live throughout their development inside the palm stem, feeding exclusively on palm tissues. This peculiar lifestyle may account for the low diversity detected in the gut of field sampled larvae of *R. ferrugineus*, regardless the investigation methods. There is strong evidence that mainly taxonomy and diet of the host can affect an organism’s gut microbial community [8,21]. RPW larvae feed on nutrient-poor palm tissues and sap that contain mainly sucrose and glucose [22] but are poor of nitrogen [20,23,24]; an excess of sugars is known to reduce the complexity of the gut microbiota [25,26]. Conversely, complex substrates, such as lignocellulose-derived materials, select complex gut bacterial communities even in highly divergent insect groups [8]. Feeding on live trees may also expose the insect to tree physiological responses, that may play a selection pressure on its microbiota. Moreover, the antimicrobial activity of larvae [27] may further shape the gut community of RPW.

The gut of RPW larvae is dominated by three phyla, Proteobacteria, Bacteroidetes and Firmicutes, that account for 98% of the assemblage. These same phyla were also found in the sugarcane weevil *Sphenophorus levis* Vaurie [28]*,* which belong to the Dryophthorinae subfamily as *R. ferrugineus,* and that is the only weevil, to our best knowledge, that has been characterized in its microbiota. Proteobacteria and Firmicutes represent also the predominant bacterial phyla in bark beetles [20] and, in general, in all insect guts studied so far, while Bacteroidetes are more prevalent in termites, detritivorous insects and, among Coleoptera, in the root feeding *Melolontha melolontha* L. (Coleoptera: Melolonthidae) [8].

The genus *Dysgonomonas* is, unexpectedly, the most represented in the gut of RPW larvae. *Dysgonomonas* (phylum Bacteroidetes) are facultative anaerobes with a fermentative metabolism producing acids and no gas, that were first recovered from a human infected gall bladder [29]. *Dysgonomonas* is described as an opportunistic human pathogen but its habitat is unknown. Members of this genus were recently detected in microbial fuel cells (MFC) anode biofilms [30], in the gut of house flies (*Musca domestica* L.) [31] and in eight separate *Drosophila* populations where its presence is not restricted to any one locality, species, or diet type [21]. Its presence in such a high number in the insect gut, and in RPW gut in particular, deserves to be further investigated because it might play an important role in the insect biology.

*Salmonella, Enterobacter, Budvicia* and other Enterobacteriaceae are highly represented in the 454 assemblage; as in other insects, they could play a beneficial role in nutrition, in the degradation of plant polymers and fermentation of sap sugars. Members of Enterobacteriaceae were also identified as intracellular symbionts of grain weevils *Sitophilus spp. *(Curculionidae) [32] and some isolates are able to fix nitrogen, thus contributing to a supplementary nitrogen source [20,23].

*Entomoplasma* is the sixth genus to be represented in terms of abundance in the RPW gut (3%). *Entomoplasma* is a glucose fermenting non-helical mollicutes and its presence in the RPW gut is consistent with what is presently known of its habitat. This genus could be considered a marker of the Coleopteran microbiota. All five currently described *Entomoplasma* species, in fact, were isolated from the gut or haemolymph of various firefly beetles (Coleoptera: Lampyridae) and green tiger beetles (Coleoptera: Cicindelidae) [33].

In spite of being affiliated to three different phyla, all the first six dominating bacterial genera of the RPW gut are facultative or obligate anaerobes with a fermentative metabolism. *Dysgonomonas* is a facultative anaerobe that produces acids and no gas; *Lactococcus* is a omofermenting lactic acid bacterium and some species can produce acetoin. The other three dominating genera belong to the Enterobacteriaceae characterized by mixed acid fermentation with production of lactic, acetic, succinic acid and ethanol (*Salmonella*), or 2,3-butanediol fermentation, producing butanediol, ethanol, CO_2_ and H_2_ (*Enterobacter* and *Budvicia*). *Entomoplasma* is also a glucose fermenting bacterium. These results suggest that the peculiar life-style of RPW larva and its gut exert a strong selective pressure towards those microbial species that are specialised to grow in a high sugar environment and that these species probably have a competitive advantage on those that cannot tolerate organic acids.

Interestingly, two genera of Enterobacteriaceae, *Pantoea* and *Rahnella,* which had previously been isolated from frass, were not detected in the gut. *Rahnella* isolates from frass have their closest relatives in components of the microbiota of the red turpentine beetle *Dendroctonus valens* LeConte (Coleoptera: Scolytidae) [20] and of the larvae of the lepidopteran *Hepialus gonggaensis* Fu & Huang (Lepidoptera: Hepialidae) [34]; *Pantoea* from frass are close to bacteria of the fungus garden of the leaf-cutter ant *Atta colombica* Guérin-Méneville (Hymenoptera: Formicidae), where they contribute to external plant biomass degradation and nitrogen fixation [35] (Additional file 5). High identities of RPW gut isolates with frass isolates and with other beneficial insect-associated bacteria suggest that the RPW gut microbiota cooperates, in a *continuum* with the frass microbiota, to the fitness of the larva inside the palm. Thus, while a unique midgut-associated microbiota can be distinguished from the environmental bacterial community in some insects [36], the peculiar lifestyle of RPW larvae makes such discrimination difficult or probably meaningless. In fact, RPW larvae feed in a very confined environment, consisting of tunnels burrowed in the palm trunk, where they continuously ingest both fresh palm tissues and frass, composed of chewed and/or digested plant tissue, so that re-acquisition by ingestion of bacteria from the environment is highly probable to occur.

Beyond nutritional aspects, the gut and frass fermentation products, such as acetoin and organic acid derivatives, ethyl esters, act as insect aggregation pheromones playing a role of attraction to other insects and promoting new oviposition events on the same tree [37].

Acidification caused by bacterial fermentation could also confer other advantages to the insect host, as some microbial toxins of Lepidoptera, such as *Bacillus thuringiensis* toxins, are activated by alkaline conditions. Thus, the RPW microbiota might help protect this insect from *B. thuringiensis* toxin by decreasing the midgut pH [38]. Moreover, together with that of fermenting yeasts, the bacterial metabolic activity increases the temperature inside the palm tissues, helping weevil overwintering [39].

It is well known that culture-based methods fail in describing the total diversity of natural bacterial communities so that, as expected, culture-independent techniques yielded a higher diversity of bacteria. Culturing under aerobic conditions led to the detection of nine bacterial genera in the RPW larval gut. Both pyrosequencing and culturing revealed that Enterobacteriaceae is the most represented bacterial family in the gut of RPW larvae.

In this work, the culture-based approach helped in obtaining a better description of some members of Enterobacteriaceae as the complete sequence of the 16S rRNA gene could be obtained from the isolated bacteria. The pyrosequencing approach, that relies upon a short 16S rRNA gene fragment, did not detect sequences of the genus *Klebsiella,* that was instead abundantly isolated by culturing. Failing of its detection could be due to low variability of the V2 region between *Klebsiella* and *Enterobacter*[12,40] and the sequences of *Klebsiella* might have been included in the genus *Enterobacter* by the RDP Classifier software.

Another genus detected by cultivation but absent in the 454 assemblage was *Bacillus* that might be present at very low levels in the RPW gut, so that its detection might be impaired by PCR biases. Bacilli isolated from the gut are close to *B. muralis* and *B. simplex,* and cluster separately from palm endophyte bacilli and frass bacilli previously isolated, that are related to the *B. cereus/thuringiensis* group.

Cuticle *Bacillus* isolates, that survived sterilization procedures, form a separate cluster from gut bacilli and are closer to the *Bacillus* isolates previously obtained from frass and from healthy palms as endophytes [2] (Additional file 5). This suggest that they belong to a bacterial community external to the larvae, that might contribute to the fitness of larvae inside the plant tissues. The cuticle aerobic spore-forming bacteria might produce antimicrobial molecules that could negatively affect the sensitivity of the larvae to entomopathogenic fungi and bacteria [41].

A low bacterial diversity and the presence of a prevailing sugar-fermenting microbiota suggest that the digestion of plant polymers (cellulose, hemicellulose) is not a primary function of the RPW larvae. However, cellulolytic and hemicellulolytic bacteria were previously isolated by enrichment cultures from the gut of RPW larvae and were mainly affiliated to the Gamma and Alphaproteobacteria of the genera *Pseudomonas*, *Enterobacter Microbacterium* and *Paenibacillus *[2]. The presence of these genera in the RPW gut was confirmed by pyrosequencing (Additional file 6). Matching the 454-reads with the 16S rRNA gene sequences of the gut cellulolytic isolates, we obtained up to 99% identity of cluster_3902 (3 sequences) with the cellulolytic isolate *Pseudomonas sp*. R-8 (Genbank accession JN167546) and 98% identity of five different clusters (for a total of 159 sequences) with the cellulolytic RPW gut isolate *Enterobacter sp*. R-10 (Genbank accession JN167548) (Additional file 6). Both isolates were positive to the Congo Red test and able to grow on xylan in pure culture [2] but their hydrolytic activity on plant polymers *in situ* has to be demonstrated (as, for example, it might be inhibited by sap sugars).

The gut of insects that rely on sugar-based diets, particularly those belonging to the orders Diptera, Hymenoptera and Hemiptera, are often dominated by acetic acid bacteria (AAB), [16]. Although the larval RPW diet is almost exclusively based on sugars, we were unable to detect AAB using a consolidated method based on the enrichment culture technique [42]. Moreover, the absence of AAB in the RPW gut was confirmed by deep sequencing, where only two sequences were affiliated to the genus *Acidisoma* (Acetobacteriaceae) (Additional file 2). AAB are common in sugary acidic and alcoholic habitats, but are usually limited by nutrients other that their primary carbon source. AAB are common in fruit-feeding *Drosophila* species but are absent in flower-feeding flies [21]. Their absence in the RPW larvae could be explained by microbial interactions occurring inside the gut. The enrichment cultures set to specifically isolate AAB led, instead, to the isolation of *Klebsiella* strains that could outcompete AABs and that could fulfil also the nitrogen fixation function [20,43], allowing the insect to live on a substrate with a high C/N ratio.

## Conclusions

The RPW microbiota is composed mainly of facultative and obligate anaerobic bacteria with a fermentative metabolism. These bacteria might have a key role in the insect nutrition, and other functions that need to be investigated. Further research, focusing on the functional traits of the bacteria inhabiting the gut of *R. ferrugineus,* is critically important to establish if some bacteria may exert an essential role for the insect or might represent an obstacle for the optimization and promotion of the use of entomopathogenic fungi and bacilli in an integrated pest management approach.

## Methods

### Sampling of RPW larvae and gut extraction

Field caught RPW late instar larvae (hereafter called larvae) were collected in Winter and Spring from infested palms of the species *Phoenix canariensis* Chabaud, located in the urban and peri-urban area of Palermo, and in San Vito Lo Capo (Trapani), (Italy) (Additional file 1). The palms were cut down following phytosanitary measures for the control and eradication of *R. ferrugineus* (Regional Decree 6 March 2007). The palms were not treated by chemical or biological pesticides.

The temperature was measured in 6 healthy and 6 infested palm trees during sampling at April 2011. Temperature was measured using a Bi-metal control digital thermometer (Wika - 360A005A4HS) by burrowing a small hole in the trunks, where the probe was inserted inside the palm trees. The average temperature of infested palm trees was 32.13°C ± 0.83, while the average temperature calculated at the same time for healthy palm trees was 25.95°C ± 0.71 and the ambient atmosphere was 26.2°C.

All sampled larvae were maintained in a plastic box with their own frass, taken from tunnels, and immediately transported to the laboratory for analysis. Each specimen was weighed, placed at -80°C for 30 min and surface sterilized with sodium hypochlorite and ethanol as described elsewhere [2,44]. Late-instar larvae (average weight = 3.5 g ± 0.7 g, body length 3 cm ± 0.6 head-capsule 6.0 mm ± 0.8), corresponding in general to the 7th instar, were used. Larvae sterilization control was performed by streaking each intact larva on the surface of a Nutrient Agar (NA, Difco) plate. Larvae were dissected, the whole gut was aseptically removed and used for DNA extraction and bacterial isolation. Each sample consisted of the content of three pooled guts extracted from three larvae of the same weight and caught at the same time in the same palm tree.

### TTGE analysis

Total bacterial diversity was assessed by Temporal Thermal Gradient gel Electrophoresis (TTGE) of 16S rDNA PCR products. DNA extraction form guts was carried out using the QIAamp DNA Stool Mini Kit, QIAGEN® (Qiagen, Hilden, Germany) according to the manufacture’s protocol and performing a lysis step at 95°C in order to obtain better lysis of Gram positive bacteria. A DNA region of approximately 200 base pairs was PCR-amplified from total DNAs. PCR was carried out using universal eubacterial oligonucleotide primers 341f-GC (5′-CGCCCGCCGCGCGCGGCGGGCGGGGCGGGGGCACGGGGGGCCTACGGGAGGCAGCAG-3′) and 534r (5′-ATTACCGCGGCTGCTGG-3′) targeting the variable V3 region of the 16S rRNA gene [45].

PCR were carried out using Phire Hot Start II DNA Polymerase (Thermo Scientific), 1X PCR buffer, 500 nM each primer, 0.20 mM dNTP and. 100 ng of DNA in a final volume of 25 μl. Cycling conditions were: 98°C for 30 sec, followed by 35 cycles of 98°C for 10 sec, 58°C for 10 sec and 72°C for 15 sec, followed by a final extension at 72°C for 2 min.

PCR products were fractionated on polyacrylamide gel (polyacrylamide:bis 29:1) 8%, Urea 7 M, Formamide 10% v/v, TAE 1.5X, at 70 V for 21 h in DCode (Bio-Rad) apparatus with a starting temperature of 57°C and a temperature ramp rate of 0.4°C h^-1^. Gels were stained with SYBRGold nucleic acid gel stain (Molecular Probes, Invitrogen) for 30 min and viewed under UV light.

Random bands were excised with a sterile scalpel immediately after visualisation, rinsed in 100 μl of distilled water and incubated in 30–50 μl of water, depending on band intensity, to elute DNA. DNA was re-amplified using the PCR-DGGE primers and products checked by agarose gel electrophoresis. The PCR products were purified using the QIAGEN PCR purification kit (Qiagen Hilden, Germany) and sequenced using the 534r primer. Partial bacterial 16S rRNA gene sequences (approximately 160 bp) were subjected to a NCBI nucleotide BLAST search (http://blast.ncbi.nlm.nih.gov/Blast.cgi) to identify sequences of the highest similarity. TTGE band sequences longer than 150 bp were deposited in Genbank under accession number KC763481 to KC763483.

### Pyrosequencing

The variable region 2 (V2) of the bacterial 16S rRNA gene was amplified with the primers 27 F (5′-AGAGTTTGATCMTGGCTCAG-3′) and 338R (5′-TGCTGCCTCCCGTAGGAGT-3′) [46], modified with Adaptor A (CGTATCGCCTCCCTCGCGCCA*TCAG*) and Adaptor B (CTATGCGCCTTGCCAGCCCGC*TCAG*), separated by the four nucleotides in italics, respectively, for pyrosequencing (Roche). The analysis was performed on DNAs extracted from a set of three larvae sampled in April 2011 (lot A) in the urban area of Palermo, Italy. PCRs for the biological samples and reagent control were carried out in five replicates with 0.6 U *Platinum*® *Taq* DNAPolymerase high fidelity (Invitrogen) in 1X PCR buffer, 2 mM MgCl_2_, 300 nM each primer, 0.24 mM dNTP and 100 ng of DNA in a final volume of 25 μl. Cycling conditions were: 94°C for 5 min, followed by 35 cycles of 94°C for 20 sec, 56°C for 30 sec and 68°C for 40 sec, followed by a final extension at 68°C for 5 min.

Equal volumes of the five reaction products were pooled and purified using the QiAquick Gel Extraction Kit (QIAGEN®). A further purification step was carried out using the Agencourt Ampure XP (Beckman Coulter Genomics), in order to obtain the required pyrosequencing-grade purity, that was assessed by loading a sample in a High Sensitivity DNA chip Agilent 2100 Bioanalyser.

PCR products were mixed for emulsion PCR at one copy per bead using only ‘A’ beads for unidirectional sequencing. Beads were subjected to sequencing on the Roche 454 GS FLX Titanium platform (Roche, Switzerland). Sequences obtained were directly clustered (no trimming was required) with CD-HIT 454 software [47] using three different similarity threshold: 90%, 95%, and 97%. This software was also used to extract representative cluster consensus sequences. After they were filtered and annotated using the Ribosomal Database Project (RDP) classifier software [48]. Filtering consisted of deleting sequences shorter than 100 bp or containing a number of unknown nucleotides (N) greater than five. Finally, all sequences (clustered plus singletons) were annotated with RDP classifier using default parameters and then parsed to obtain a readable text file in output.

The most abundant unique sequence of each OTU cluster (family or, when possible, species) was selected as representative, then aligned by SINA [49], mounted in ARB [50] and subjected to chimera check (before submission in GenBank) by Pintail v. 1.1 software [51]. Rarefaction curves were generated from families of clustered OTUs using EcoSim v.1.2d [52], separately for each percentage of similarity.

The 97% similarity clustered consensus sequences were deposited in Genbank under accession numbers KC896717-KC896758; raw reads were deposited in NCBI Sequence Read Archive with accession number SRR837401 (reference: BioProject PRJNA196888).

### Bacterial isolation and identification

The aerobic cultivable bacterial fraction was analysed on three lots of larvae sampled in April 2011 in Palermo (lots A, B, C). Each pool consisted of three larval guts and their total average weight was 3.68 g (SD: 0.18).

RPW guts were aseptically extracted from each larva, then the content of three guts was pooled, serially diluted in sterile physiological solution, and plated on NA. The plates were incubated for 72 h at 28°C. At the end of the incubation period, colonies were counted and single colonies were streaked to purity on the same fresh medium. The isolates were grouped into OTUs by ARDRA analysis. The whole 16S gene was amplified by colony PCR using the bacterial universal primers fD1 and rD1 [53], as described elsewhere [2], and the amplicons were digested using the restriction enzymes *AluI* and *AfaI*. Representative isolates of each OTU were randomly chosen for bidirectional sequencing of the 16S rRNA gene. Colonies growing on sterilization control plates were streaked to purity and analysed by ARDRA and 16SrRNA gene partial sequencing. In the same time enrichment cultures in a sorbitol-containing medium at pH 3.5 were set as described by Yamada et al. [42], for the isolation of acetic acid bacteria (AAB). When microbial growth occurred, the microorganisms were streaked on CaCO_3_ agar plates and colonies capable of causing clearing of the CaCO_3_ were selected and identified by partial sequencing of PCR-amplified 16SrRNA gene. Sequences were subjected to NCBI nucleotide BLAST search as described above. Amplified sequences and close relatives were aligned using SILVA alignment tool [54]. Alignment was merged with SSUref_108_Silva_NR database and manually checked with ARB [50]. After alignment, the neighbour-joining algorithm of ARB package was used to generate the phylogenetic trees based on distance analysis for 16S rRNA genes. The robustness of inferred topologies was tested by bootstrap re-sampling using the same distance model (1000 replicates). 16S rRNA gene sequences were deposited in Genbank under accessions number KC584753 to KC584772 (gut isolates), KC763479-80 (cuticle isolates) and KC763478 (AAB enrichment culture isolate).

### Addendum

Recently, just before this manuscript was submitted to this journal, a study on the seasonal variation of the intestinal metagenomes of *R. ferrugineus* larvae and adults from date palms was published [55]. This study reports that, at the phylum level, Proteobacteria dominate the gut metagenomes of date palm larvae, followed by Tenericutes or Firmicutes depending on the season. The authors identify *Klebsiella pneumoniae* and *Lactococcus lactis* as the dominant species of the microbiota. Bacteroidetes are found at negligible levels and the genus *Dysgonomonas* is not detected. Differences between larvae from date palm and those from Canary palm may be attributed to the host plant species.

The metagenomic analysis carried out by Jia et al. [55] describes a few ORFs involved in the hydrolisis of cellulolose and high abundance of ORFs related to utilization of disaccharides and simple sugars that are abundantly available in the palm sap [24]. Sap sugars are presumably the main C and energy source for the RPW larvae and its microbiota, that is dominated by fermenting bacteria to obtain several metabolites including lactate and acetate.

## Competing interests

The authors declare that they have no competing interests.

## Authors’ contributions

MT projected and carried out the microbiological and molecular analyses, EM performed the bioinformatic analyses, BM identified and collected the insects in the field and manipulated them for the gut microbiota analyses, SC constructed the phylogeny trees and helped to draft the manuscript, PQ conceived and coordinated the study and drafted the manuscript. All authors read and approved the final manuscript.

## Supplementary Material

Additional file 1 ***Phoenix canariensis *infested by *Rhynchophorous ferrugineus *(A and B); different infested palms cut in the higher part are shown.** Larvae of the red palm weevil (RPW) *Rhynchophorus ferrugineus*, found inside the body of the infested palm (C). Female adult specimen of *Rhynchophorus ferrugineus* Olivier (Coleoptera, Curculionidae, Rhynchophorinae) (D). Click here for file

Additional file 2 Complete results of 16S pyrotag sequence clustering and taxonomic assignment by RDP of clusters and singletons at 90%, 95% and 97% ID. Click here for file

Additional file 3 Relative abundance of bacterial families in the gut of RPW larvae as detected by pyrosequencing of the 16SrRNA gene V2 region. Click here for file

Additional file 4 **Phylogenetic tree of 16S rRNA gene amplicons clustered at 97% consensus.** The tree was constructed by neighbour-joining method and Jukes Cantor distance matrix using the arb software. Bootstraps were calculated over 1000 random repetitions: values >60 and < =75 are shown as open circles, whereas values >75 are shown as filled circles. Sequences obtained in this study are indicated in bold. The scale bar represents 10% sequence divergence. Click here for file

Additional file 5 **Phylogenetic tree of 16S rDNA sequences of RPW gut isolates and related sequences, as determined by distance Jukes-Cantor analysis.** One thousand boostrap analyses were conducted and values greater than 60% are reported. Two Archaea sequences of *Methanopirus kandleri* and *Korarchaeum cryptophilum* were used as outgroup. The scale bar represents the expected number of changes per nucleotide position. Colors indicate the isolation site or the isolation procedure described in this work and in [2]. Blue: RPW gut isolates on NA; Red: frass bacteria; Green: palm bacterial endophytes; Fuchsia: gut isolates obtained from enrichment cultures on CMC; Yellow: larval cuticle bacteria isolated from sterilization control plates. Isolate_RPWenrichAAB* was isolated from the RPW larval gut from enrichment cultures set for for Acetic Acid Bacteria isolation [42]. Click here for file

Additional file 6 **Identities of 16S rRNA gene sequences of cellulolytic bacterial strains isolated from the RPW gut by enrichment cultures **[2] **with the RDP 454 consensus sequences clustered at 97% ID of the RPW microbiota.** Only identities above 90% are shown. Click here for file
