# Supplementary figures and images for: The gut microbiota of larvae of Rhynchophorus ferrugineus Oliver (Coleoptera: Curculionidae)
Source: BMC Microbiol. 2014 May 30;14:136. doi: 10.1186/1471-2180-14-136 (PMC4060583; doi:10.1186/1471-2180-14-136)

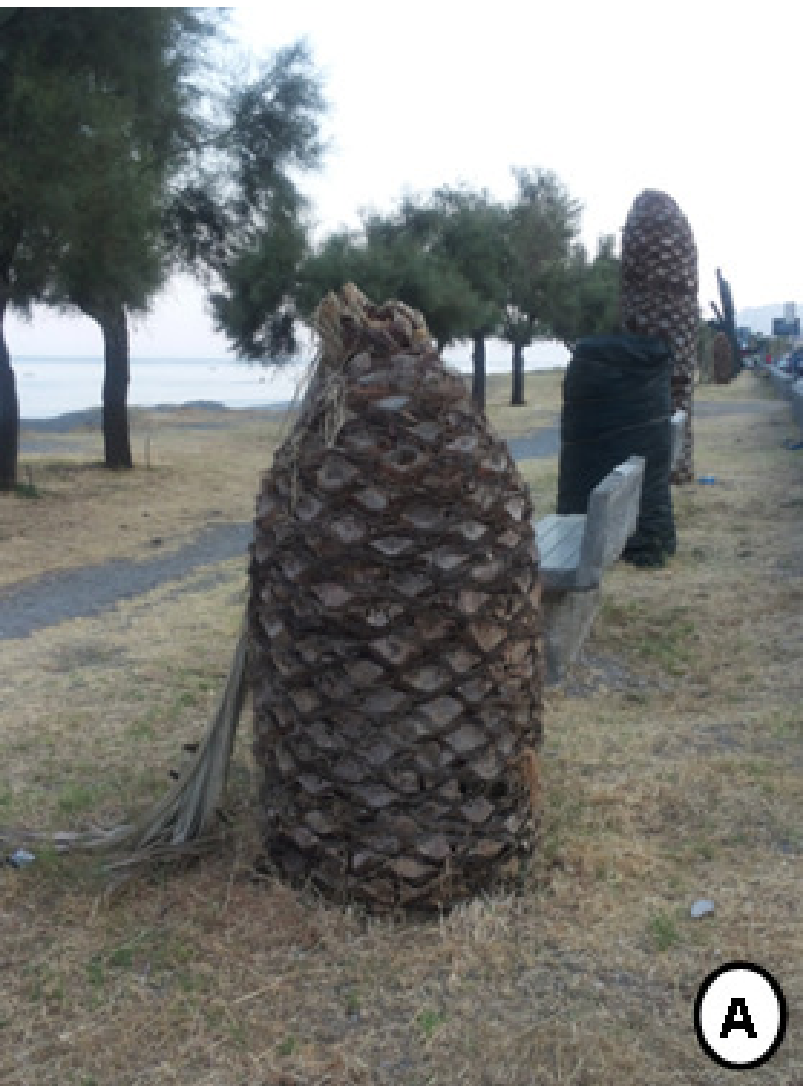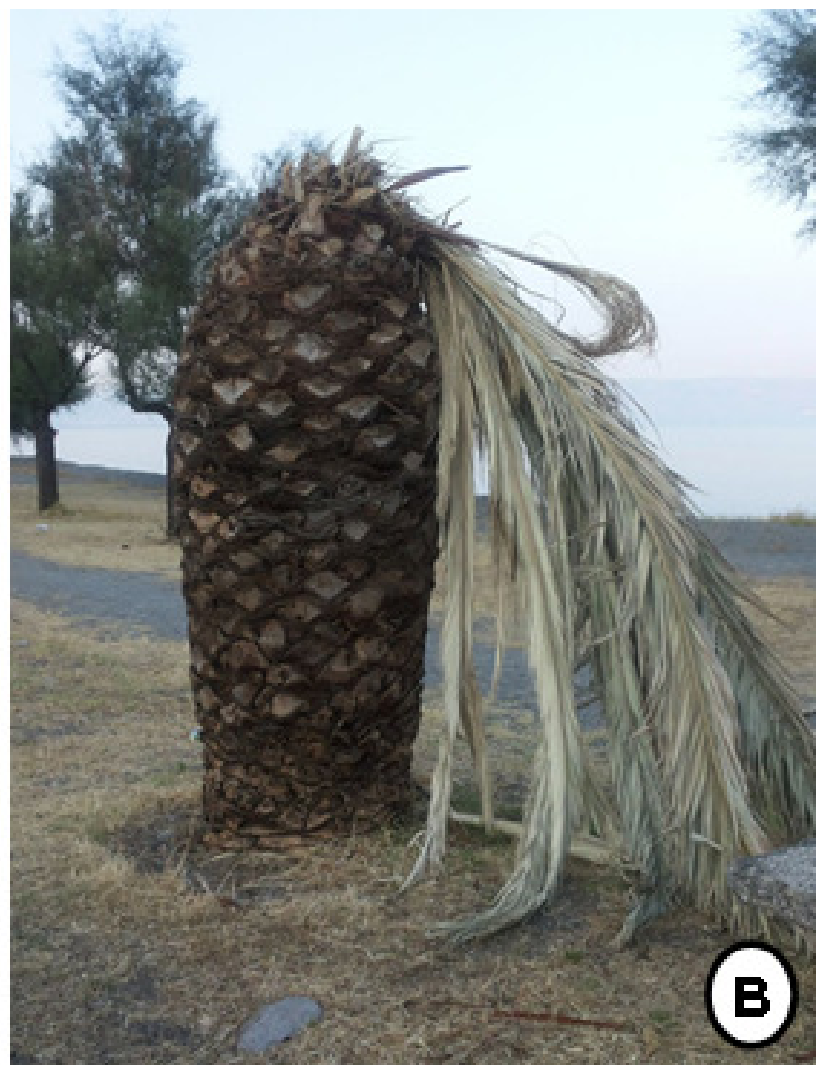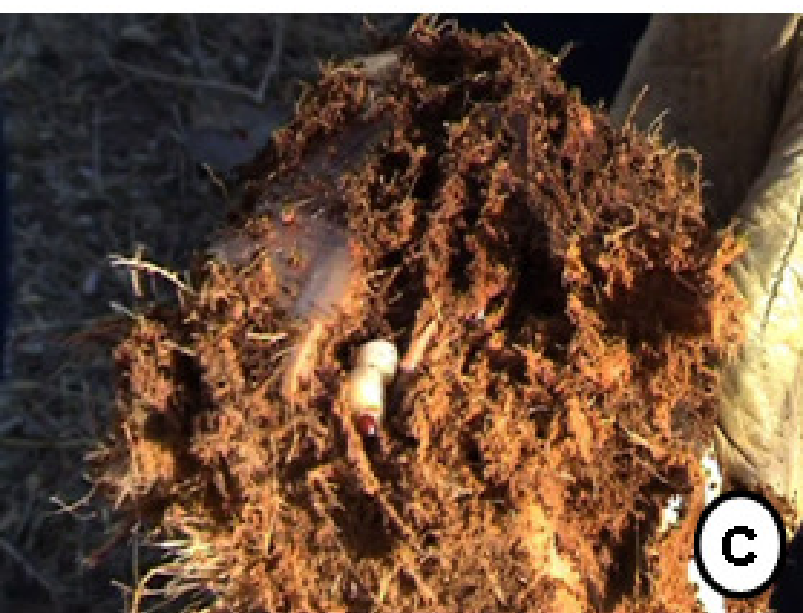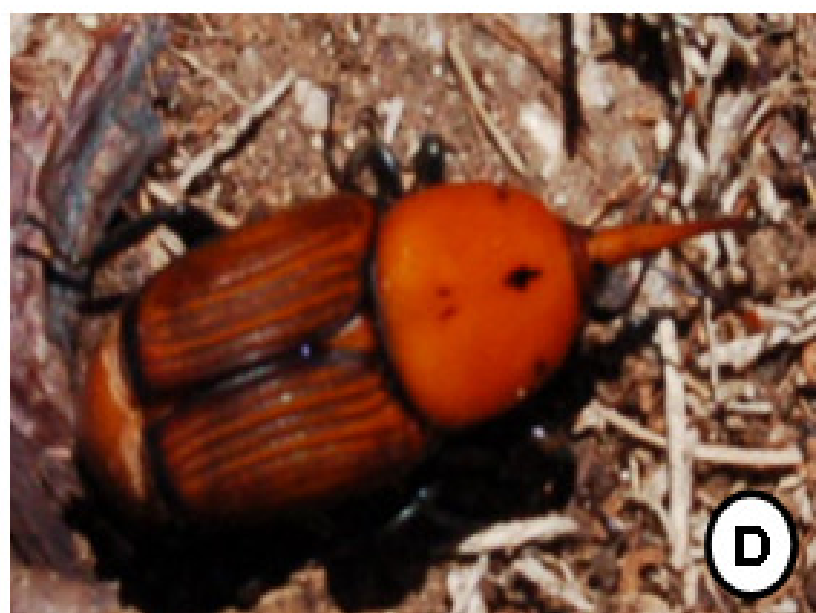

Supplement: Additional file 1 — Phoenix canariensis infested by Rhynchophorous ferrugineus (A and B); different infested palms cut in the higher part are shown. Larvae of the red palm weevil (RPW) Rhynchophorus ferrugineus, found inside the body of the infested palm (C). Female adult specimen of Rhynchophorus ferrugineus Olivier (Coleoptera, Curculionidae, Rhynchophorinae) (D). [file 1471-2180-14-136-S1.pdf]

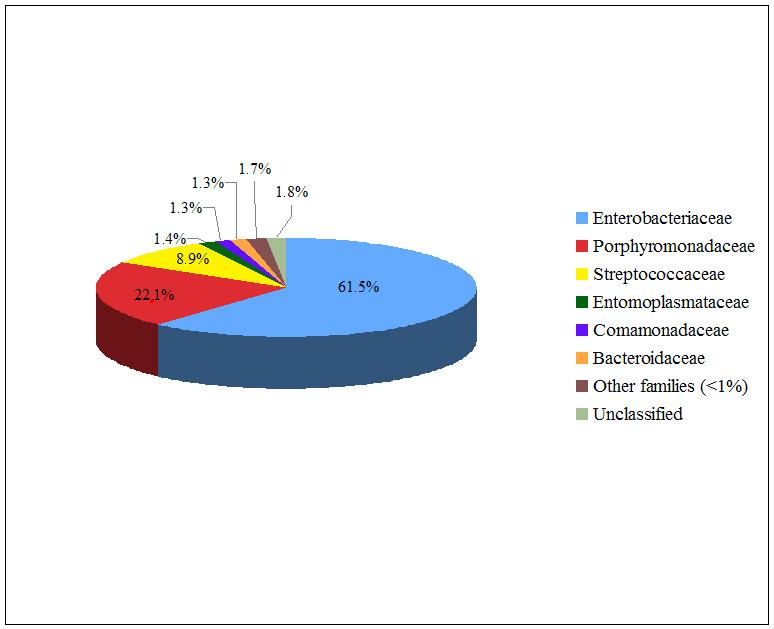

Supplement: Additional file 3 — Relative abundance of bacterial families in the gut of RPW larvae as detected by pyrosequencing of the 16SrRNA gene V2 region. [file 1471-2180-14-136-S3.jpeg]

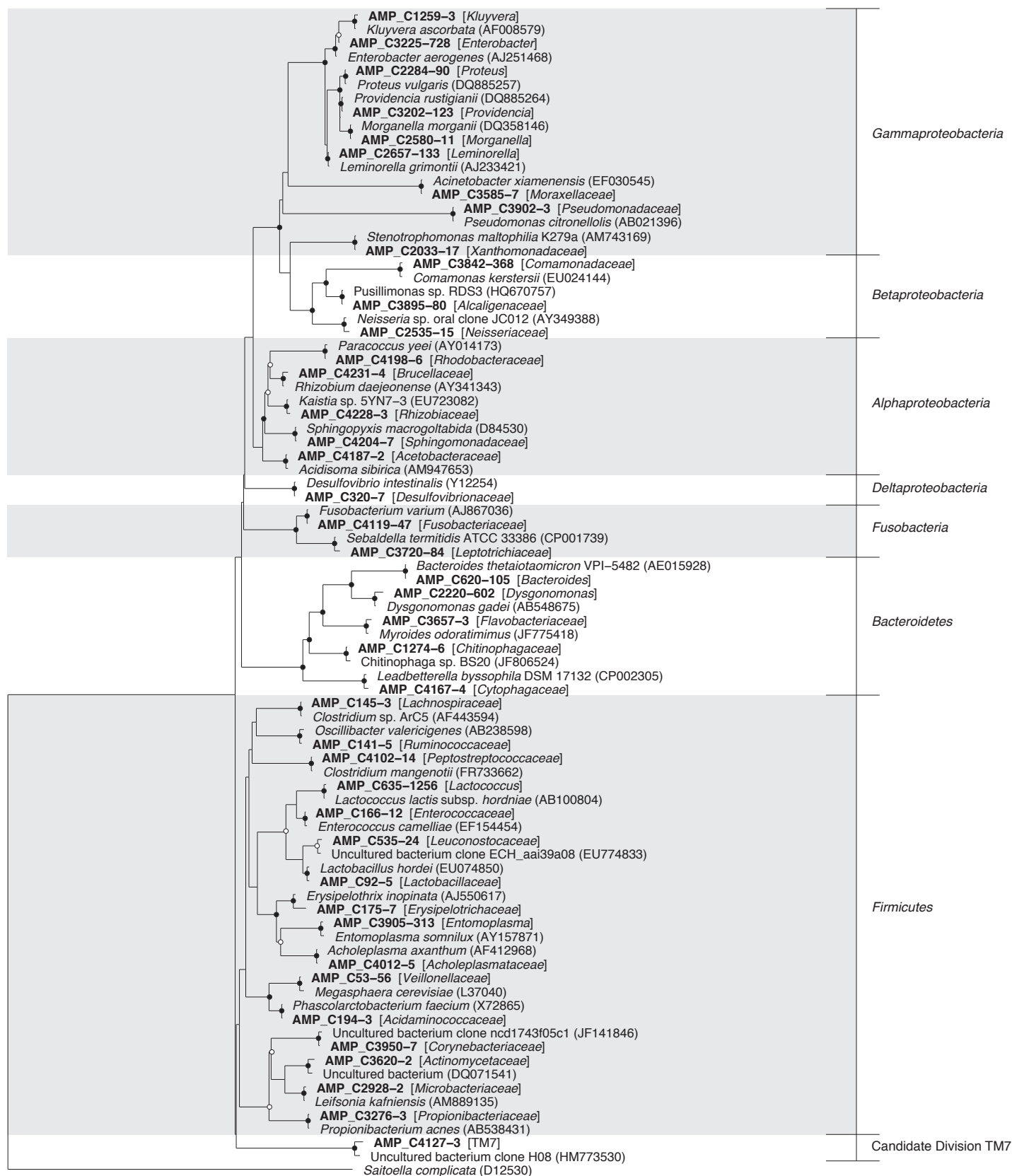

0.10

Bootstrap (n=1000), % strength

- 60-75%
- >75-100%

Supplement: Additional file 4 — Phylogenetic tree of 16S rRNA gene amplicons clustered at 97% consensus. The tree was constructed by neighbour-joining method and Jukes Cantor distance matrix using the arb software. Bootstraps were calculated over 1000 random repetitions: values >60 and < =75 are shown as open circles, whereas values >75 are shown as filled circles. Sequences obtained in this study are indicated in bold. The scale bar represents 10% sequence divergence. [file 1471-2180-14-136-S4.pdf]

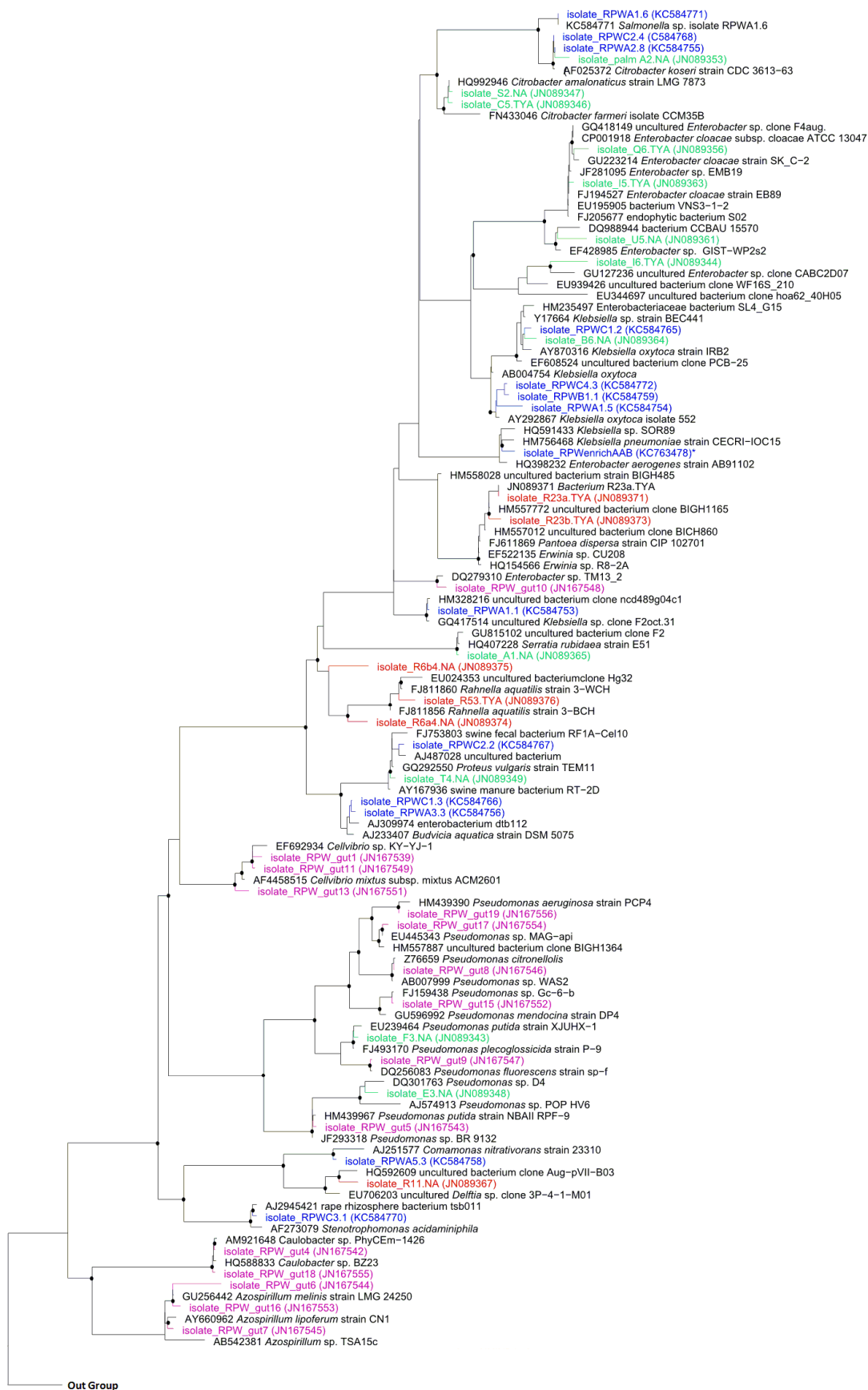

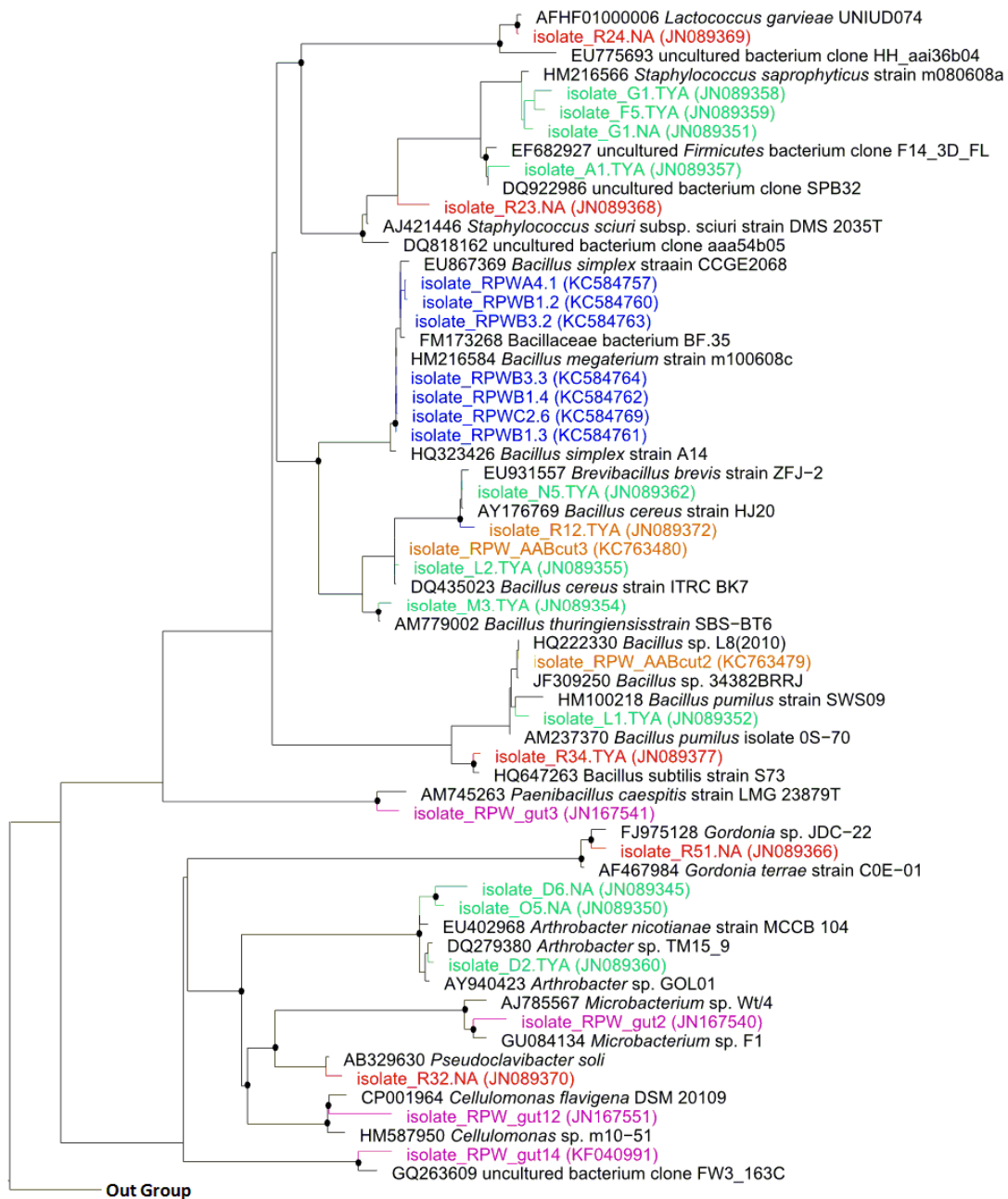

Supplement: Additional file 5 — Phylogenetic tree of 16S rDNA sequences of RPW gut isolates and related sequences, as determined by distance Jukes-Cantor analysis. One thousand boostrap analyses were conducted and values greater than 60% are reported. Two Archaea sequences of Methanopirus kandleri and Korarchaeum cryptophilum were used as outgroup. The scale bar represents the expected number of changes per nucleotide position. Colors indicate the isolation site or the isolation procedure described in this work and in [2]. Blue: RPW gut isolates on NA; Red: frass bacteria; Green: palm bacterial endophytes; Fuchsia: gut isolates obtained from enrichment cultures on CMC; Yellow: larval cuticle bacteria isolated from sterilization control plates. Isolate_RPWenrichAAB* was isolated from the RPW larval gut from enrichment cultures set for for Acetic Acid Bacteria isolation [42]. [file 1471-2180-14-136-S5.pdf]
