# Supplementary material for: The gut microbiota of larvae of Rhynchophorus ferrugineus Oliver (Coleoptera: Curculionidae)
Source: BMC Microbiol. 2014 May 30;14:136. doi: 10.1186/1471-2180-14-136 (PMC4060583; doi:10.1186/1471-2180-14-136)
Supplement: Additional file 6 — Identities of 16S rRNA gene sequences of cellulolytic bacterial strains isolated from the RPW gut by enrichment cultures [2] with the RDP 454 consensus sequences clustered at 97% ID of the RPW microbiota. Only identities above 90% are shown. [file 1471-2180-14-136-S6.pdf]

Additional file 6

Table. Alignments of 16S rRNA gene sequences of cellulolytic bacterial strains isolated from the RPW gut by enrichment cultures on cellulose (Butera et al., 2012) and the 97% RDP 454 consensus sequences of the RPW microbiota. Only identities above 90% are shown.

**RPW cellulolytic Isolate**	**Genbank Accession Number**	**BLASTN best match RDP_Consensus_97% (number of sequences)**	**Identity % (nt/nt)**	**Score (bits)**	**E-value**
Pseudomonas sp R-5	JN167543	cluster_3902 (3)	95 (304/317)	518	1e-147
		singleton_cluster_3928	92 (296/319)	459	7e-130
		cluster_3626 (4)	90 (291/322)	416	4e-117
Pseudomonas sp R-8	JN167546	cluster_3902 (3)	99 (315/317)	579	5e-166
		singleton_cluster_3928	94 (303/319)	497	1e-141
		singleton_cluster_2983	93 (303/324)	475	6e-135
Pseudomonas sp R-9	JN167547	cluster_3902 (3)	93 (274/292)	436	3e-123
		singleton_cluster_2983	92 (236/256)	363	6e-101
		singleton_cluster_3928	90 (266/293)	387	3e-108
Pseudomonas sp R-15	JN167552	cluster_3902 (3)	93 (277/297)	436	2e-123
		singleton_cluster_3928	91 (272/298)	399	3e-112
Enterobacter sp R-10	JN167548	cluster_3487 (15)	98 (259/264)	460	2e-130
		cluster_3410 (30)	98 (259/264)	460	2e-130
		cluster_3264 (76)	98 (259/264)	460	2e-130
		cluster_3184 (21)	98 (259/264)	460	2e-130
		cluster_3104 (17)	98 (259/264)	460	2e-130

BLASTN 2.2.24+

Reference: Zheng Zhang, Scott Schwartz, Lukas Wagner, and Webb Miller (2000), "A greedy algorithm for aligning DNA sequences", J Comput Biol 2000; 7(1-2):203-14.

Database:RDP_Consensi_97.fa 3,863 sequences; 1,289,650 total letters.
